# Limited evidence of patient-to-patient transmission of *Staphylococcus aureus* strains between children with cystic fibrosis, Queensland, Australia

**DOI:** 10.1371/journal.pone.0275256

**Published:** 2022-10-07

**Authors:** Sharon L. Biggs, Amy V. Jennison, Haakon Bergh, Rikki Graham, Graeme Nimmo, David Whiley

**Affiliations:** 1 School of Medicine, The University of Queensland, UQ Centre for Clinical Research (UQCCR), Herston, Queensland, Australia; 2 Public and Environmental Health, Forensic and Scientific Services, Coopers Plains, Queensland, Australia; 3 Pathology Queensland Central Laboratory, Herston, Queensland, Australia; 4 School of Medicine, Griffith University, Gold Coast, Queensland, Australia; The Rockefeller University, UNITED STATES

## Abstract

**Objectives:**

Here we used whole genome sequencing (WGS) to understand strain diversity and potential for patient-to-patient transmission of *Staphylococcus aureus* among children with cystic fibrosis (CF) in Queensland, Australia.

**Methods:**

*S*. *aureus* isolates (n = 401) collected between January 2018 and April 2019 from 184 patients with CF (n = 318 isolates) and 76 patients without CF (n = 83 isolates) were subjected to WGS and subsequent multilocus sequence typing (MLST), and a phylogeny was constructed from core genome single nucleotide polymorphism (SNP) analysis. The subsequent data was compared with available patient information.

**Results:**

WGS revealed that patients with CF were essentially colonised by the same genotypes as those seen in patients without CF. Sequence types (ST) for our patients with CF were predominantly ST5 (20.1%), ST30 (7.3%), ST15 (6.3%) and ST8 (5.3%). Two Australian clones, ST93 and ST239, typically seen in skin infections and health-care settings, respectively, were notably absent from our patients with CF. Based on a SNP distance threshold of 14 SNPs, 20 cluster types involving 50/260 patients were evident; of these, 6 clusters contained only patients found to be siblings or otherwise living in the same household. Epidemiological relationships could not be determined for a remaining 14 cluster types involving 38 patients, comprising 2–7 (median 2) patients each. Multiple *S*. *aureus* genotypes were observed in 19/73 CF patients who provided more than one sample.

**Conclusion:**

These results show that WGS is a useful tool for surveillance of *S*. *aureus* strains in children with CF and that the strains in our CF cohort were largely consistent with those circulating in patients without CF. Overall, this confirms previous findings and indicates that *S*. *aureus* acquisition in children with CF is similar to that of other patient groups, with limited evidence of potential patient-to-patient transmission within this patient group.

## Introduction

Cystic fibrosis (CF) is a multi-system autosomal recessive genetic disorder characterised by numerous abnormalities including chronic pulmonary infections, increased sweat, malabsorption and other gastrointestinal challenges [1]. The underlying pathophysiology of pulmonary infections is impaired ion transport in airway and lung epithelium that causes fluid hyperabsorption, imbalanced osmotic pressure and hyperconcentrated mucus. This results in airway obstruction and impaired mucocilliary clearance of the airways of patients with CF, establishing a niche for opportunistic colonising bacteria that often progresses to infection [2]. Worldwide, including in Australia, the most prevalent infecting pathogens in patients with CF include *Pseudomonas aeruginosa*, methicillin-resistant and methicillin-susceptible *Staphylococcus aureus* (MRSA and MSSA, respectively), *Haemophilus influenza*, *Stenotrophomonas maltophilia*, *Burkholderia cepacia complex* and *Aspergillus spp*. [3, 4]. For over sixty years, *P*. *aeruginosa* and *S*. *aureus* have been associated with CF [5]. *P*. *aeruginosa* and MRSA, in particular, are associated with lung function decline, increased exacerbations, increased morbidity and mortality, and overall reduced rates of survival of patients with CF [2]. Early infection with these microbes of the airways and lungs of these patients can lead to irreversible damage to the airway surfaces, with adverse long-term consequences. The epidemiology of *P*. *aeruginosa* strains in patients with CF has been extensively explored globally, including in Australia [6, 7]. It is now well-recognised that there are particular *P*. *aeruginosa* strains shared among patients with CF, including LES, Clone C and the Australian AUST-01 and AUST-02 and AES-1, AES-2 and AES-3 strains, and that some of these shared strains are associated with increased morbidity and mortality [6, 8]. However, few studies have examined dissemination of *S*. *aureus* strains in patients with CF [9–16], and questions remain over the potential for shared strains of *S*. *aureus* among this patient group, particularly among children with CF.

While *S*. *aureus* is often a commensal organism, it is also one of the first pathogenic bacteria to invade and infect the lungs of children with CF [17]. Colonising *S*. *aureus* in the upper airways of people with CF are a reservoir for lower airway infection [17] and, once a child’s lungs are infected, eradication can be difficult. *S*. *aureus* is highly transmissible and acquisition can occur by contact with contaminated surfaces or colonised or infected individuals, including patients, health care workers, the general public or animals [18, 19]. Here, using whole genome sequencing (WGS), we sought to investigate the potential for shared *S*. *aureus* strains within a paediatric CF population in Queensland, Australia. The Queensland Children’s Hospital is the largest Cystic Fibrosis Service for children with CF in Australia. Awareness and prevention measures, such as the National Hand Hygeine Initiative [20] and infection control information and procedures from Cystic Fibrosis Australia [21], have helped reduce the spread of *S*. *aureus* in the CF and wider communities. There remains, however, strong representation of *S*. *aureus* in children with CF in Queensland [3]. Regular screening and surveillance of pathogens in children with CF is key to guiding practices aimed at reducing transmission and the overall morbidity and mortality of this vulnerable population. Hence, these results could potentially improve understanding of management of *S*. *aureus* infection in children with CF.

## Methods

### Samples

Here, 401 isolates were processed through the Microbiology Department, Central Laboratory, Pathology Queensland, at the Royal Brisbane Women’s Hospital (RBWH), Herston, between January 2018 and April 2019. All samples were collected from patients aged up to 18 years at the date of collection, including 318 isolates from 184 patients with CF and 83 isolates from 76 patients without CF (a convenience collection of isolates from patients with *S*. *aureus*-related clinical conditions were included to serve as a reference bank). Patients with CF attended the Queensland Children’s Hospital (new patients under 16 years of age; continuing patients to 18 years of age) and patients without CF attended various hospitals throughout Queensland. Between 1 and 7 samples were collected per patient, which are described in Table 1.

**Table 1:** Summary of isolates.

	Patients with CF (PC) n (%)		Patients without CF (PN) n (%)		Total n (%)
Isolates	318 (79.3)		83 (20.7)		401 (100.0)
Patients	184 (70.8)		76 (29.2)		260 (100.0)
Females	82 (44.6)		31 (40.8)		113 (43.5)
Males	102 (55.4)		45 (59.2)		147 (56.5)
**Age ranges**	Males n (%)	Females n (%)	Males n (%)	Females n (%)	
<2	14 (4.4)	9 (2.8)	8 (9.6)	11 (13.3)	42 (10.5)
2–5	37 (11.6)	21 (6.6)	11 (13.3)	5 (6.0)	74 (18.5)
6–11	64 (20.1)	54 (17.0)	17 (20.5)	8 (9.6)	143 (35.7)
12–18	66 (20.8)	53 (16.7)	15 (18.1)	8 (9.6)	142 (35.4)
**Sample types**					
Swab/sputum	310 (97.5)		1 (1.2)		311 (77.6)
BAL	6 (1.9)		3 (3.6)		9 (2.2)
Aspirate	2 (0.6)		7 (8.4)		9 (2.2)
Swab–other^a^	0		66 (79.5)		66 (16.5)
Other^b^	0		6 (7.2)		6 (1.5)
**Isolates/patient**					
1	111 (42.7)		69 (26.5)		180 (69.2)
2	40 (15.4)		7 (2.7)		47 (18.1)
3	18 (6.9)		0		18 (6.9)
4	7 (2.7)		0		7 (2.7)
5	4 (1.5)		0		4 (1.5)
6	3 (1.2)		0		3 (1.2)
7	1 (0.4)		0		1 (0.4)

^a^ Swab—other includes abscess, skin, lesion and wound sites.

^b^ Other include pus, tissue, bone marrow.

Isolates were coded by a unique S sample number, and each patient by a unique P identifier (ID) (PC for patients with CF and PN for patients without CF). Lowercase alphabetic character suffixes to P IDs denote multiple samples provided by that patient. Roman numeral suffixes represent different colonies from the same bead stock from the same patient.

### Routine testing

Processing of isolates was performed according to standard protocols as part of routine testing utilising the Vitek®MS (bioMérieux) and the Vitek®2 XL (bioMérieux) for species identification and antimicrobial susceptibility testing, respectively. Vitek®2 antimicrobial susceptibility testing for *S*. *aureus* included penicillin G (β-lactamase), oxacillin, gentamicin, ciprofloxacin, erythromycin, clindamycin, linezolid, daptomycin, teicoplanin, vancomycin, tetracycline, nitrofurantoin, fusidic acid, mupirocin, rifampicin and trimethoprim-sulfamethoxazole (SXT). Inconclusive cefoxitin/oxacillin screen results were supplemented with Oxoid^TM^ Cefoxitin antimicrobial susceptibility disc testing (Thermo Scientific^TM^) on Mueller-Hinton agar and reported according to EUCAST [22]. Presence of the *mec*A gene was analysed on select isolates by *S*. *aureus* Staphylococcal chromosomal cassette (SCC) *mecA* real-time PCR.

### Isolate preparation for whole genome DNA sequencing

An aliquot from frozen bead stock was used to inoculate 5% horse blood agar (HBA—Edwards) plates. When smaller and scant colonies were observed, species identification was confirmed by Pastorex™ Staph Plus (Bio-Rad) latex agglutination and multiple aliquots were used to inoculate HBA and/or mannitol salt agar (MSA–in-house) plates. After 24 hours incubation in CO_2_/O_2_, respectively, at 35°C, a single colony was selected from these plates to inoculate a new 5% HBA plate, which was incubated for 24 hours under the same conditions. After inspection, this plate was referred for WGS at the Public Health Microbiology Laboratory, Forensic and Scientific Services (FSS), Queensland Health.

Prior to whole genome sequencing, species identification was reconfirmed by MALDI-TOF MS, using IVD-CE MALDI Biotyper (Bruker) at FSS.

### Whole genome sequencing

WGS was performed at the Queensland Health FSS laboratory using standard protocols and quality control procedures. DNA was extracted from isolates using the QIAsymphony® DSP DNA Mini Kit (Qiagen, Germany) and fragmented and tagged for sequencing using the Nextera® XT DNA Library Prep Kit(Illumina®,CA, USA). Solid phase reversible immobilisation (SPRI) library cleanup was performed on a Zephyr® (Perkin Elmer, UK) and libraries were quantified using the 2200 TapeStation (Agilent, USA). DNA libraries were sequenced on the NexSeq 500 sequencer (Illumina, CA, USA) using the NexSeq 500 Mid Output v2.5 Kit (300 cycles) (Illumina®) according to the manufacturer’s instructions.

Sequences were trimmed for quality using Trimmomatic v0.32 [23] and assembled using *SPA*des Assembler v3.12.0 [24]. Quality assessment used FastQC v0.11.5 (Babraham Bioinformatics) and MultiQC v1.1 (https://multiqc.info/). Sequences were considered to pass QC parameters if they were at least 130bp in length after trimming and gave at least 40x coverage of the *S*. *aureus* genome. Assemblies were considered to pass QC if there were fewer than 500 contigs and the total contig length was as expected for *S*. *aureus* (approximately 2.8Mb). Quality data for each isolate was recorded, assessed and accepted, or the WGS was repeated.

#### Genotype analysis

All genotypic analyses were performed on WGS data. Genotypic analysis involved screening for acquired antimicrobial resistance, virulence factors and multilocus sequence type (MLST) identification, using databases via ABRicate v2 [25] (accessed 17/10/2019). Resistance databases, NCBI [26], CARD [27] and Resfinder v3.1 [28] used default parameters of 75% identity and 0 coverage. VFDB [29] (accessed 26/01/2021) with default parameters of 90% identity and 50% protein coverage, and MLST [25] (accessed 28/10/2019), screened for virulence factors and MLST respectively. Screening for acquired antimicrobial resistance, as well as resistance-associated point mutations, used NCBI’s AMRfinderPlus v3.9 software [26, 30] (accessed 14/4/2021). Clonal complex (CC) and MLST (ST) analysis utilised pubMLST [31] and the BIGSdb [32]. *Spa* typing used Ridom SeqSphere+ v7.2 [33] (accessed 3/3/2021). Accessory gene regulator (*agr*) typing used AgrVATE v1.0 [34] (accessed 5/5/2021).

Genomic identification of SCC*mec* was performed using SCC*mec*Finder [35–38]. Mapping/confirmation of SCC*mec*, including *mecA*, *mecB*, *mecC* and *mecD*, and *bla* operons, used Geneious Prime v2020.1 (https://www.geneious.com).

#### Phylogenetic analysis

The Nullarbor v2.0 [39] pipeline for public health microbiology was used for phylogenetic analysis of WGS data. Nullarbor utilises Kraken v1.1.1 for species identification. SNP distances, produced by phylogenetic analysis that focuses on variation in the core genome, can be used to identify clonal groupings of genome sequences to determine epidemiological distribution and possible transmission events [40–42]. Nullarbor utilised Snippy v4.4 [43] for core SNP alignment. Post-recombination predicted phylogeny was produced using Gubbins v2.4.1 [44] for recombination detection based on regions of increased SNP-density and SNP-sites v2.5.1 [45] for removal of these regions from the core SNP alignment. Maximum likelihood phylogeny was predicted using FastTree v2.1.10 [46] and IQ-TREE v1.6.12 [47] with ModelFinder [48] and UFBoot [49] (1000 bootstrap replicates) options. A matrix of pairwise SNP distances between each isolate to analyse their genomic relatedness was produced by SNP-dists v0.7.0 (https://github.com/tseemann/snp-dists), using the post-recombination phylogenetic data. We have described isolates, with the same patient ID, from the same patient, as “linked”, and isolates from different patients, with different IDs as “unlinked”. Reference strain for SNP analysis was NZAK3 (NCBI Accession NZ_LT009690.1), selected using kmerFinder v3.1 [50–52]. Tree data from Nullarbor and IQ-TREE was imported into Interactive Tree of Life iTOL v4 [53] to draw phylogenetic trees.

### Statistical analysis

Statistical analysis was performed using Fisher exact test at https://www.socscistatistics.com/tests/fisher/default2.aspx.

### Ethics approval

This study was approved in writing by Children’s Health Queensland Hospital and Health Service Human Research Ethics Committee—approval number HREC/17/QRCH/100. Consent was waived by the ethics committee and all data was fully anonymized prior to accessing same.

## Results

Approximately 3500 Australians have CF, 46% of whom are under 18 years of age and around 1 in 25 people in Australia are purported to be carriers of gene mutations that cause CF. Of the 880 people in Queensland with CF in 2019, children account for 51%, or approximately 450 [54].

Table 1 provides a summary of all isolates in the study. In brief, 401 *S*. *aureus* isolates from 260 patients with *S*. *aureus*, that were collected over a 16-month period, were sequenced. These isolates included 318/401 (79.3%) from 184/260 (70.8%) patients with CF and 83/401 (20.7%) from 76/260 (29.2%) patients without CF. Isolates from patients with CF included 82/184 (44.6%) females and 102/184 (55.4%) males. Isolates from patients without CF included 31/76 (40.8%) females and 45/76 (59.2%) males. Samples from patients with CF were mostly cough swab/sputum (n = 310/318, 97.5%) with the remainder being aspirates or bronchiolar lavage (BAL) (n = 8/318, 2.5%). Samples from patients without CF included one cough swab/sputum (n = 1/83, 1.2%), with the majority being wound/abscess swabs (66/83, 79.5%) or tissue or bone marrow (n = 16/83, 19.2%). Between 1 and 7 samples were collected per patient (mean 1.54 samples/patient) and 80/260 (30.8%) patients provided more than one isolate. 73/184 patients with CF (39.7%) provided 207/318 isolates (65.1%) and 7/76 patients without CF (9.2%) provided 14/83 isolates (16.9%).

For the purpose of analysis, duplicates were removed by grouping isolates with the same patient ID and ST classification as one case (n = 287, 207/287 for patients with CF, 80/287 for patients without CF).

### Phylogenetic analysis

**Fig 1:**
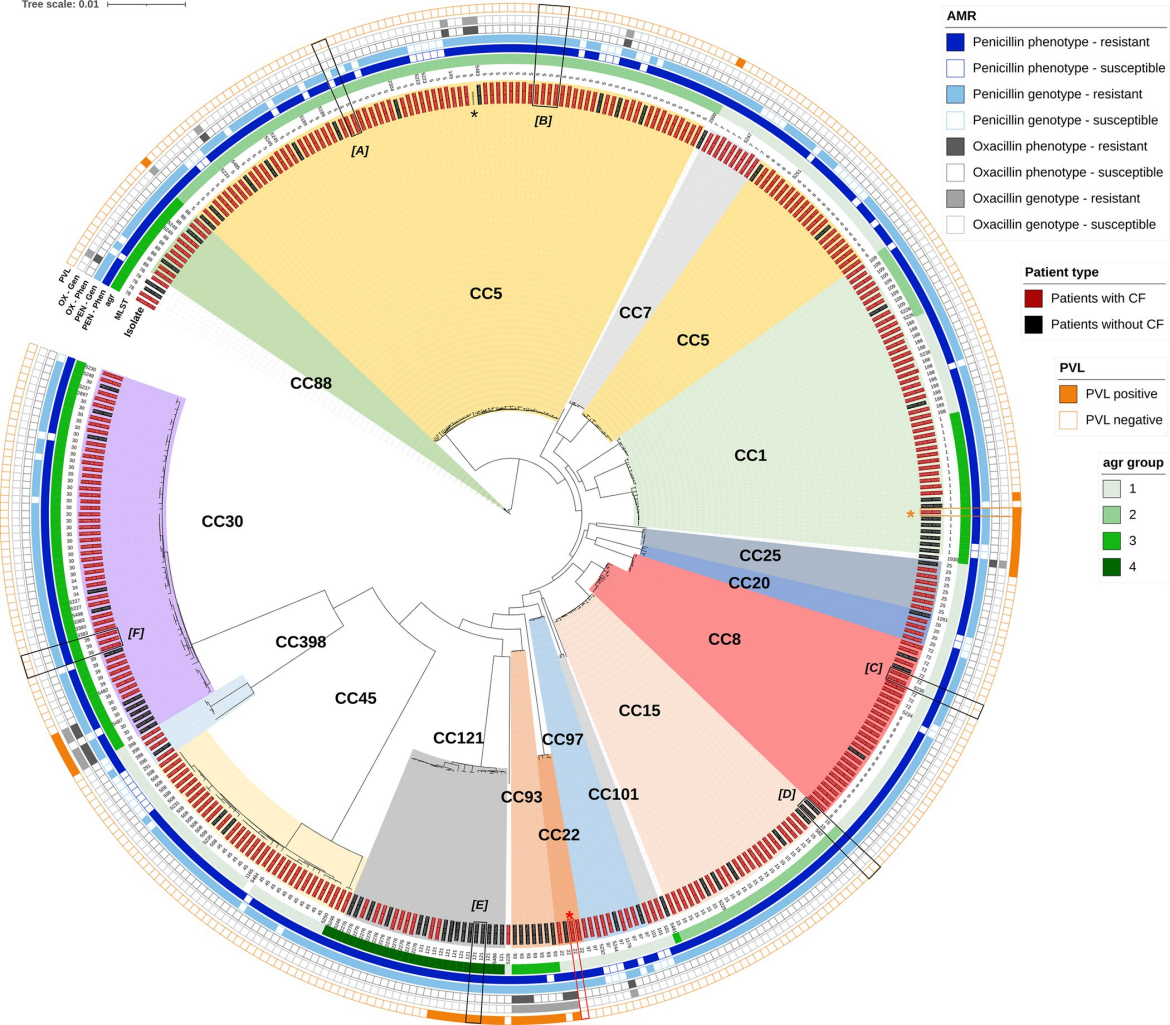
Circular phylogram of *S*. *aureus* isolates from patients with and without CF.

**Fig 2:**
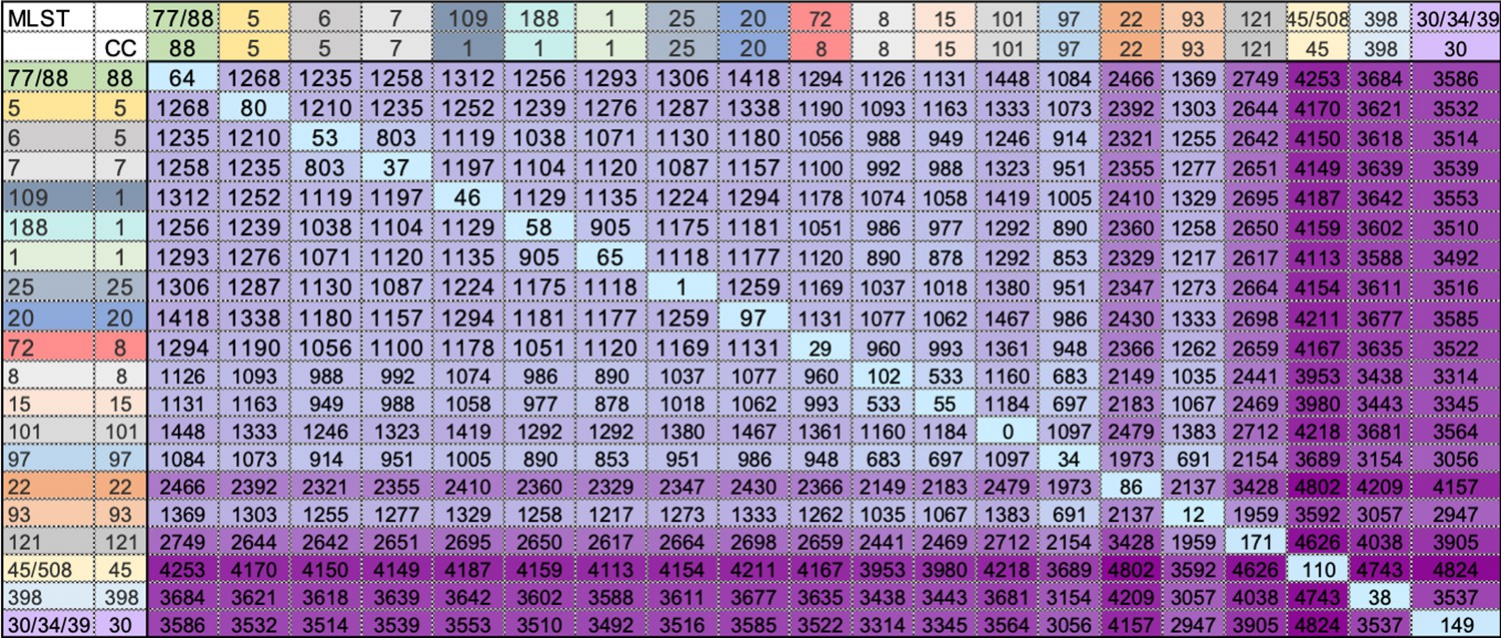
Heat map of median pairwise SNP distances between major clades.

WGS data and associated phylogenetic analyses by core SNPs for all isolates are presented in Fig 1 and summarised in Fig 2 (and further detailed in S2 Table). Data presented in Fig 1 include CC and their respective MLST, agr type, phenotypic and genotypic penicillin and oxacillin resistance profiles and PVL presence or absence. STs not designated to CCs by pubMLST were assigned based on publications [14, 55].

Circular phylogram showing *S*. *aureus* isolates from patients with and without CF distributed within the major Clonal Complexes (clades), their respective MLSTs (innermost ring) and *agr* types (second outer ring), phenotypic and genotypic penicillin and oxacillin resistance profiles (third, fourth, fifth and sixth rings, respectively) and PVL presence/absence (outermost ring). Boxes: black—linked isolates *[A]* PC0063 and PC0100, *[B]* PC0092 and PC0094, *[C]* PC0147 and PC0221, *[D]* PN0219 and PN0220, *[E]* PN0235 and PN0259 and *[F]* PC0038 and PC0187; orange*—PVL positive patient with CF; red*—EMRSA-15 (PC0048a). black*—reference strain. CC—clonal complex, MLST—multilocus sequence type, PEN–penicillin, OX–oxacillin, Phen–phenotype, Gen–genotype, PVL–Panton-Valentine Leukocidin, R–resistant, S–sensitive, pos–positive, neg–negative.

Median pairwise SNP distances within and between the major clades of the phylogenetic tree in Fig 1. SNP distances: within clades (light blue) ranged from 0 to 215 (median 83), between clades (purple) ranged from 478 to 4875 (median 2629). Heat map colours: darker represents larger SNP distances, lighter represents smaller SNP distances. MLST—multilocus sequence type, CC—clonal complex.

Overall, the *S*. *aureus* isolates’ SNP-derived clusters aligned with CC clades and their respective MLST subclades. CCs 5, 30, 1, 45, 8, and 15 make up approximately 74% (n = 211/287) of total cases, including 79% (n = 164/207) of cases from patients with CF and 58% (n = 47/80) of cases from patients without CF. Cases from patients with CF were significantly more likely to have CC5 (n = 57/207 27.54%, n = 13/80 16.25%, respectively, *p* = .0476) and CC45 (n = 20/207 9.66%, n = 2/80 2.50%, respectively, *p* = .0470). Conversely, cases from patients without CF were significantly more likely than patients with CF to be infected with CC121 (n = 14/80 17.50%, n = 7/207 3.38%, respectively, *p* = .0001) and CC93 (n = 7/80 2.44%, n = 0/207 0%, respectively, *p* = .0001).

The seven most commonly detected sequence types in our patients with CF were ST5 (CC5), ST30 (CC30), ST1 and ST188 (CC1), ST45 (CC45), ST8 (CC8) and ST15 (CC15), which, when combined, constituted approximately 53% (n = 110/207) of cases with CF and 49% (n = 141/287) of total cases. ST5 accounted for over 20% of cases for patients with CF (n = 43/207), 10% for patients without CF (n = 8/80) and 17% (n = 51/287) of total cases. Certain STs were mostly observed among patients with CF, including ten cases of ST45 (n = 10/207, 4.83%) that were present only in patients with CF. Conversely, ST93 consisted solely of seven cases of patients without CF (n = 7/80, 8.75%).

*Agr* and *spa* typing were also performed for these isolates and are included in S1 Table. The *agr* types strongly correlated with STs, with *agr* types 1, 2, 3 and 4 comprising 33.4%, 31.4%, 27.2% and 7.7% of cases respectively. In contrast, *spa* types were considerably more diverse with multiple *spa* types detected for each MLST (e.g. up to 20 different types for ST5), and some *spa* types were shared between STs. The most common *spa* type was t002 (n = 17/287, 5.9%), 94% (n = 16/17) of which were from cases with CF, including 14 (n = 14/17, 82.4%) with ST5 (CC5). S2 Table details isolates’ CC, MLST, *agr* and *spa* types.

### Population structure by SNPs

SNP distance typing was used for inference of genomic relatedness between linked (same patient) and unlinked (different patient) isolates to more reliably investigate the possibility of transmission events within this cohort [56–58]. Fig 2 shows the median pairwise SNP distances within (light blue) and between (purple) the major clades in the phylogenetic tree that range from 0 to 4875 (median 2305). SNP distances ranged from 0 to 215 (median 83) within and from 478 to 4875 (median 2629) between clades. For example, the SNP distances of CC5 clades, ST5 and ST6, range from 0 to 133 (median 80) and 0 to 82 (median 53), respectively. However, between these clades SNP distances range from 1184 to 1236 (median 1210). In comparison, the pairwise SNP distances within CC45 clade (ST45 and ST508) range from 0 to 149 (median 110) and have a median SNP distance of 4170 from the more distantly-related ST5 cluster. Clustering of isolates from the same patient (i.e. linked) on neighbouring tree branches of the phylogenetic tree, and belonging to the same CC and/or ST, produced 71 cluster types with SNP distances that ranged from 0 to 14 (median 2). This enabled us to define a genomically-related based SNP distance threshold of 14 SNPs.

**Fig 3:**
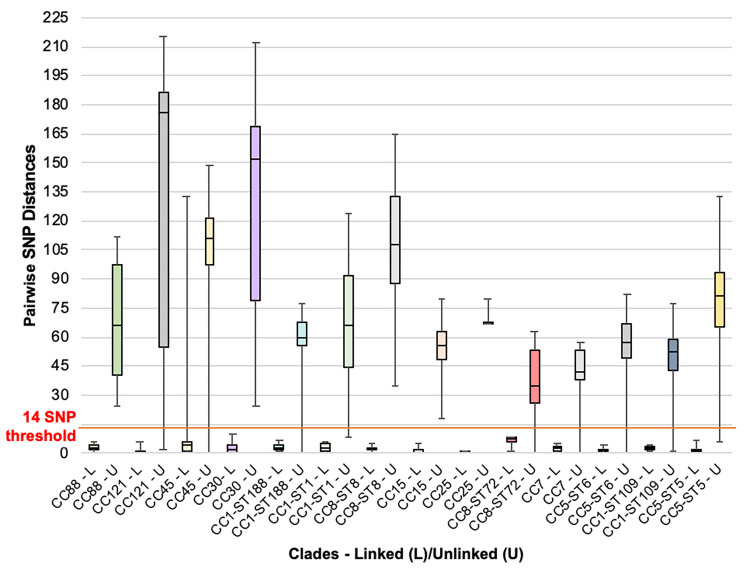
Pairwise SNP distances within the major clades for linked and unlinked isolates from all patients.

Fig 3 demonstrates the SNP distance threshold by comparing SNP distances of linked (L) and unlinked (U) isolates for the major clades of the phylogenetic tree. Two examples are patients PC0018 and PC0095 of CC5 and CC45, respectively. PC0018 samples, S0018, S0101, S0163, S0298, all strain CC5-ST5-*agr*2-t2396, with pairwise SNP distances between 0 and 2, median 1, plotted in the upper quartile of the CC5-ST5-L (linked) box; and PC0095 samples, S0135, S0095, S0109, S0235, S0246, all CC45-ST508-*agr*1 with SNP distances between 4 and 6, median 4, plotted in the upper quartile of the CC45-L (linked) box. However, pairwise SNP distance between PC0105 isolates (both CC45-ST508-*agr*1) of 133 extends the whisker beyond this threshold.

Pairwise SNP distances within the major clades for linked and unlinked cluster types that plotted below and above the 14 SNP distance threshold, respectively. CC—clonal complex, ST–multilocus sequence type, L—linked, U–unlinked.

Having established the 14 SNP distance threshold for epidemiologically-linked isolates (based on multiple isolates from individual patients), we then applied this threshold the entire cohort to determine if any isolates from unlinked patients showed genomic clustering. This identified 20 cluster types, involving 50 unlinked patients (PC n = 29/184, PN n = 21/76, 17.4% cases) with SNP distances below the 14 SNP distance threshold, represented by the bottom whiskers of the unlinked boxes for each clade in Fig 3. Investigation into these isolates was conducted based on available information, including name, age, date of birth, address (all of which were coded and de-identified for the purposes of this study), and dates of current and previous sample collections, to reveal 12 patients (n = 12/287, 4.2%) with epidemiological associations that represent probable transmission events. These are shown in the phylogram (Fig 1) enclosed in black boxes and include patients who are most probably siblings with the same surname and address, including PC0038 and PC0187, PC0092 and PC0094, PC0147 and PC0221, PN0235 and PN0259, and PN0219 and PN0220; and patients PC0063 and PC0100 residing in the same household. No obvious epidemiological association based on the level of the current investigation was found between the remaining 13 cluster types (n = 36/287, 12.5% cases). These remaining cluster types included eight involving only patients with CF (two patients each, n = 16/38), two involving only patients without CF (n = 13/38) and three involving patients with and without CF (PC n = 4/38, PN n = 3/38).

### Patients with more than one sample

**Fig 4:**
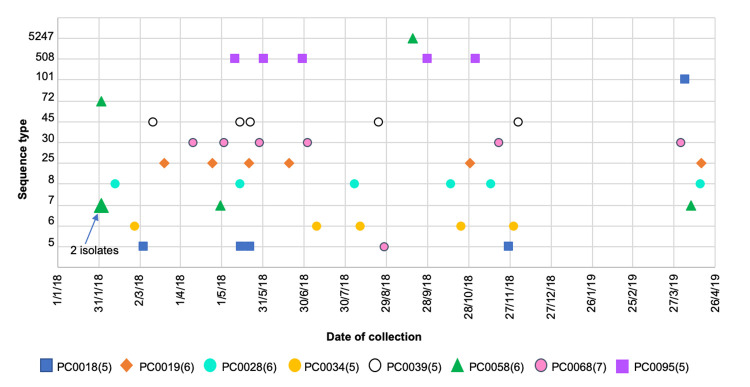
Colonising sequence types from patients with five or more isolates tested.

Persistence of *S*. *aureus* in the respiratory tract and lungs of patients with CF, has been previously demonstrated and, especially in the case of MRSA infections, results in worsening outcomes for these patients, including lung function decline and respiratory tract inflammation [59–61]. Most often, one predominant strain takes up long-term residence. However, the sequential or simultaneous presence of more than one strain in some patients has also been reported [62]. Of the 73 patients with CF who provided more than one isolate (n = 80/260), only one ST was observed in 54/73 (74.0%) patients, two different STs were observed in 15/73 (20.5%) patients and three different STs in 4/73 (5.5%) patients. This is illustrated in Fig 4 where isolates from seven patients with CF, each with more than five samples, are plotted by their STs and dates of collection. Two and three genetically-distinct isolates were detected in patients PC0018 and PC0058, respectively.

Isolates from patients who provided five or more samples, by MLST and date of collection. Patients (n = number of samples). Each marker represents one isolate, except where two isolates are indicated for PC0058. PC–patients with CF.

The presence of more than one strain was detected in the same sample for four patients (represented by patient IDs with roman numeral suffixes). When slower-growing, pin-point colonies were observed on agar plates in the laboratory, indicating possible small colony variants (SCVs), multiple agar plates were inoculated using aliquots from the same bead stock and submitted for sequencing. The same ST was detected in three isolates from PC0179 and two different STs were detected from each of four patients, PC0058, PC0177, PN0200 and PC0267. This is also demonstrated in Fig 4 for PC0018 in February, 2018.

### Resistance to beta-lactams

As with MLST analysis, duplicates were removed by grouping isolates with the same patient ID, ST classification and β-lactam resistance phenotype as one case (n = 306, 226 for patients with CF, 80 for patients without CF). The majority of cases were phenotypically resistant to penicillin (n = 270 88.2%), with no significant differences observed between patients with CF (n = 199/226 88.1%) and without (n = 71/80 88.8%). Phenotypic resistance to oxacillin was observed in 16 (5.2%) cases overall. However, significantly fewer instances were observed for patients with CF (n = 4/226 1.8%) compared to those without CF (n = 12/80 15.0%, *p* < .0001). Resistance to β-lactams is shown in Fig 1.

*S*. *aureus* methicillin resistance genes are generally harboured by the SCC*mec* mobile element, with 14 different types, I to XIV, currently recognised [63]. Only one isolate from a single sampling event was determined to be MRSA from patient PC0048 with CF, who provided multiple samples. SCC*mec* type IV was detected in isolates PC0048a, PC0075 and PC0212 and SCC*mec* type V in PC0202.

PC0048a was EMRSA-15 SCC*mec*IVh ST22-2A clone, characterised by the presence of *sbi*, *seg*, *sei*, *sak*, *scn*, *chp*, S80F and S84L mutations in topoisomerase IV and gyrase A genes, respectively, that confer ciprofloxacin resistance, *cap5H*, *-I*, -*J*, *-K* genes, T69 nucleotide deletion in *ureC* gene, and SNPs, as described previously [55, 64]. However, *seb*, *ermC* and *entC* genes were absent, and *spa* type t3287 replaced t032 in PC0048a.

In addition to the 12 patients without CF who harboured MRSA, five MSSA isolates from as many patients also harboured a SCC*mec* operon encoding methicillin resistance genes. All 17 patients harboured CA-MRSA SCC*mec* type IV.

### Panton Valentine Leukocidin (PVL)

PVL is a bi-component, pore-forming virulence factor of *S*. *aureus* encoded by the *lukF-PV* and *lukS-PV* genes [65] that is commonly associated with CA-MRSA and present in pandemic strains such as USA300 (ST8) and ST93 [66]. Overall, 11.8% (n = 34/287) of cases in our cohort harboured PVL genes, as seen in Fig 1. These included 33 patients without CF, 14 of whom harboured SCC*mec* type IV MRSA and one patient with CF, PC0065, with MSSA CC1-ST1-agr3-t127. Strain types belonged to CC121 (n = 9/34), CC1 (n = 9/34), CC93 (n = 7/34), CC30 (n = 5/34) and CC96, CC22, CC25 and CC5.

## Discussion

Prior to the 1990s, *P*. *aeruginosa* strains harboured by patients with CF were thought to be unique and predominantly acquired from the environment, and, apart from sibling transmission, the sharing of strains was considered rare [8, 67]. It is now, however, accepted that some *P*. *aeruginosa* strains are clonal in nature and may be shared extensively among patients within the same CF clinic [67], or, in some instances, between clinics due to the movement of patients across geographical regions [68]. Currently, there are no reports of routinely-collected data regarding strains of *S*. *aureus* detected in patients with CF in Australia, and publications in this respect are rare globally. Transmission of *S*. *aureus* occurs in both hospital and community settings and may occur via environmental sources [69], animals [70], health care workers and other patients [71]. Apart from the presence of the CF-Marseille lineage reported amongst two CF centres between 2002 to 2006 in France [72], *S*. *aureus* strains identified in patients with CF are reported to largely reflect locally-circulating epidemic lineages and strains, and so acquisition is considered for the most part to be by independent colonisation events [11, 57].

Overall, the results of our study are consistent with the above. Of the total 184 patients with CF studied, we found evidence of strains potentially being shared among a total of 26 patients, comprising two patients in each of 13 cluster types. Notably, four (n = 4/13) cluster types could be readily explained as siblings, or otherwise residents of the same household. Households are known reservoirs for transmission of *S*. *aureus* and transmission between siblings is common among patients with and without CF [57, 73]. We did, however, identify eight clusters (n = 16 patients, two patients per cluster) involving patients with CF for which transmission could not be explained. Without supporting epidemiological links (such as temporal or geographical, that would be present in an outbreak), or follow-up investigation, we are hesitant to conclude that these do indeed represent patient-to-patient transmission. SNP distance analysis alone is not sufficient to conclude transmission [74], but these clusters warrant further investigation.

The WGS data also provided a useful snapshot of circulating clones and sequence types among our cohort. While many strains present in the respiratory samples of children with CF in Queensland were also present in various sample types from children without CF, patients with CF in Queensland did not harbour all major strains shown to be circulating in the wider population. Of note, one patient with CF harboured epidemic clone EMRSA-15 and two widely-circulating strains associated with invasive infections in Queensland and Australia [75], ST239 and ST93, were rare in our cohort.

EMRSA-15 is an international pandemic clone with origins in the UK that was first detected in Australia in 1997 [76]. Since its peak as the predominant Australian hospital-associated (HA-MRSA) clone in 2014, incidence of EMRSA-15 has declined from 29% to 16.4% in 2019, when it was exceeded only by ST93-IV, that accounted 24.5% of Australia’s MRSA [75]. HA-MRSA ST22-IV(2B) in Australia is PVL-negative and 97.8% ciprofloxacin and 65.2% erythromycin non-susceptible, consistent with PC0048a, which was PVL-negative and ciprofloxacin-resistant, but erythromycin-sensitive.

ST93 [77] is a highly virulent and dominant community-associated strain in Australia, mostly attributed to skin infections, but also associated with necrotising pneumonia in otherwise healthy individuals and almost invariably carries PVL [78]. ST93 accounted for 24.4% of cases of invasive CA-MRSA infections in Australia and 40.4% in Queensland in 2019 [78], but was notably absent in our patients with CF. Likewise, ST239 is an international epidemic clone, with multi-resistant, hospital-associated Australian sub-clones Aus-2 and Aus-3 [55]. While its prevalence in Australia and Queensland has dramatically reduced since 2003 [79], ST239 still accounted for 50.0% of HA-MRSA invasive infections in Queensland in 2019, therefore, its prevalence in patients without CF in our cohort was markedly low. Furthermore, although ST239 has also been linked with patients with CF overseas [11, 14, 80], it was not detected in any children with CF in our Queensland cohort. Interestingly, the rarer ST398 [81] strain was detected in two children with CF. ST398 is a multi-resistant, livestock-associated strain of *S*. *aureus* that is strongly associated with animal contact [82, 83] and not easily transferred between humans [55]. CA-MRSA ST398 is currently a dominant strain in China [84], but has rarely been reported in Australia and, similar to ST239, has been reported in very few patients with CF globally [15].

The presence of MRSA in our cohort was relatively low, with only 3.4% of patients with CF harbouring MRSA strains. This is consistent with the rate of 5.7% MRSA carriage in patients with CF under 18 years of age in Australia [3]. Likewise, few PVL-positive strains were identified. Colonisation and invasion by PVL-positive MSSA and MRSA occurs more often in otherwise healthy, immunocompetent individuals in the absence of risk factors, and is associated with skin and soft tissue infection and necrotising, but not hospital-acquired, pneumonia [85]. The health consequences of *S*. *aureus* PVL-positive strains in patients with CF remain varied [86, 87]. As PVL carriage was only detected in one patient with CF, our findings are in line with reports that PVL is less often associated with patients with CF and more often associated with skin and soft tissue infections in patients without CF [86–88].

The fact that patients with CF may be simultaneously infected by multiple *P*. *aeruginosa* strains, including mixtures of shared strains, is well-documented, and has been demonstrated in young patients with CF [68, 89]. Mixed strains of *S*. *aureus* in patients with CF have also been reported [62, 90]. Numerous factors may contribute to the presence and detection of multiple strains in the airways and lungs of patients with CF, including the introduction of a new strain by way of transmission, within-host evolution as a strain adapts by mutation or gene acquisition to environmental pressures [90–92] and immune system evasion strategies such as conversion to small colony variants in the presence of biofilm [61, 91, 93]. In our cohort of children with CF, more than one genotype was observed in 19 of the 73 patients with multiple samples. On the basis of SNP distances and ST, co-colonisation of distantly-related isolates most probably represent the acquisition of a new strain. The presence of multiple genetically-related strains, however, may represent evolving strains that have adapted to the unique CF environment. Adaptation and persistence in terms of antimicrobial resistance and virulence genes will be the focus of further investigation.

There are limitations to this study, including our reliance on routine collection of patient samples. Therefore, for some patients, the disparate sampling in terms of numbers of samples collected and/or tested, may have lead to an underpresentation of *S*. *aureus* diversity and/or transmission. Although our study comprised a relateivly large cohort of patients with CF, there were considerably fewer isolates from patients without CF. Likewise, there were some biases associated with our comparisons between CF and non-CF groups, noting that the isolates from the latter group were primarly from skin infections. Some discrepancies between phenotypic and genotypic resistance to β-lactams were evident, which may reflect alternative mechanisms of resistance, or a limitation of isolating and testing only 1 colony from a potentially heterogeneous population of *S*. *aureus* that has adapted to the lungs and airways of a patient with CF. However, as the focus of this paper was the epidemiology of *S*. *aureus*, the topic of resistance will be further explored in future publications. Also, as our ethical approvals extended only to relatively limited, routinely-collected patient information, the potential transmission events outlined above could not be more thoroughly investigated.

## Conclusion

Overcoming the challenge of *S*. *aureus* infections in patients with CF requires a greater understanding of circulating strains and transmission thereof. Our data highlights potential utility of whole genome sequencing in this regard and the potential for *S*. *aureus* SNP distance analysis in surveillance for *possible* transmission events. Specifically, our data show that there were no shared strains of note in our population and that patient-to-patient transmission, outside of patients living within the same household, is relativey rare.

## Supporting information

S1 Table List of patient sample data. List of patient samples including clonal complex, MLST, *agr* and *spa* types, penicillin and oxacillin susceptibility, PVL presence or absence.
(DOCX)

S2 Table Summary of major clonal complexes. MLST, *agr* and *spa* types and number of cases for the major clonal complexes.
(DOCX)
