# Supplementary material for: Limited evidence of patient-to-patient transmission of Staphylococcus aureus strains between children with cystic fibrosis, Queensland, Australia
Source: PLoS One. 2022 Oct 7;17(10):e0275256. doi: 10.1371/journal.pone.0275256 (PMC9543978; doi:10.1371/journal.pone.0275256)
Supplement: S1 Table — List of patient samples including clonal complex, MLST, agr and spa types, penicillin and oxacillin susceptibility, PVL presence or absence. (DOCX) [file pone.0275256.s001.docx]

**Title:** Limited evidence of patient-to-patient transmission of *Staphylococcus aureus* strains between children with cystic fibrosis, Queensland, Australia.

**Journal:** PLOS ONE

**Authors**: Sharon L Biggs^1^, Amy V Jennison^2^, Haakon Bergh^3^, Rikki Graham^2^, Graeme Nimmo^4^, David Whiley^1,3*^

^1^School of Medicine, The University of Queensland, UQ Centre for Clinical Research (UQCCR), Herston, Queensland 4029, Australia; ^2^Public and Environmental Health, Forensic and Scientific Services, Coopers Plains, Queensland 4108, Australia; ^3^Pathology Queensland Central Laboratory, Herston, Queensland, 4029, Australia; ^4^School of Medicine, Griffith University, Gold Coast, Queensland 4215, Australia.

*Corresponding author: Assoc Professor David Whiley, The University of Queensland, UQ Centre for Clinical Research (UQCCR), Herston, Queensland 4029, Australia.

Email: [d.whiley@uq.edu.au](mailto:d.whiley@uq.edu.au)

Phone: +61 7 3346 5053

**Supplementary Information SI1**

**Table SI1** List of patient samples including Clonal Complex, MLST, *agr* and *spa* types, penicillin and oxacillin susceptibility, PVL presence/absence

| **Patient-Sample** | **CC** | **MLST** | ***agr*** | ***spa*** | **Penicillin (R/S)** | **Oxacillin (S/R); [SCCmec type]** | **PVL +/-** |  |
| --- | --- | --- | --- | --- | --- | --- | --- | --- |
| PC0001a-S0001 | 88 | 78 | 3 | new | R | S | - |  |
| PC0001b-S0173 | 88 | 78 | 3 | new | R | S | - |  |
| PC0002a-S0002 | 15 | 15 | 2 | t346 | R | S | - |  |
| PC0002b-S0166 | 15 | 15 | 2 | t346 | R | S | - |  |
| PC0002c-S0200 | 15 | 15 | 2 | t1877 | R | S | - |  |
| PC0003a-S0003 | 398 | 398 | 1 | t1184 | R | S | - |  |
| PC0003b-S0067 | 398 | 398 | 1 | t1184 | R | S | - |  |
| PC0004a-S0004 | 5 | 5 | 2 | t045 | S | S | - |  |
| PC0004b-S0104 | 5 | 5 | 2 | t002 | S | S | - |  |
| PC0004c-S0279 | 5 | 5 | 2 | t062 | S | S | - |  |
| PC0005a-S0005 | 1 | 1 | 3 | t14508 | R | S | - |  |
| PC0005b-S0195 | 1 | 1 | 3 | t14508 | R | S | - |  |
| PC0006-S0006 | 15 | 15 | 2 | t12818 | R | S | - |  |
| PC0007a-S0007 | 5 | 5 | 2 | t002 | R | S | - |  |
| PC0007b-S0188 | 5 | 5 | 2 | t002 | R | S | - |  |
| PC0007c-S0216 | 5 | 5 | 2 | NT | R | S | - |  |
| PC0008-S0008 | 20 | 20 | 1 | t164 | R | S | - |  |
| PC0009a-S0009 | 1 | 5226 | 2 | t209 | R | S | - |  |
| PC0009b-S0171 | 1 | 5226 | 2 | t209 | R | S | - |  |
| PC0010-S0010 | 1 | 1 | 3 | t127 | R | S | - |  |
| PC0011-S0011 | 5 | 5 | 2 | new | S | S | - |  |
| PC0012-S0012 | 8 | 8 | 1 | t121 | R | S | - |  |
| PC0013-S0013 | 15 | 15 | 2 | t605 | R | S | - |  |
| PC0014-S0014 | 30 | 30 | 3 | t4733 | R | S | - |  |
| PC0015a-S0015 | 5 | 5 | 2 | t010 | R | S | - |  |
| PC0015b-S0087 | 5 | 5 | 2 | t010 | R | S | - |  |
| PC0015c-S0278 | 5 | 5 | 2 | t653 | R | S | - |  |
| PC0016-S0016 | 15 | 5229 | 2 | t085 | R | S | - |  |
| PC0017a-S0017 | 101 | 101 | 1 | NT | S | S | - |  |
| PC0017b-S0158 | 101 | 101 | 1 | t2078 | S | S | - |  |
| PC0018a-S0018 | 5 | 5 | 2 | t2396 | R | S | - |  |
| PC0018b-S0101 | 5 | 5 | 2 | t2396 | R | S | - |  |
| PC0018c-S0163 | 5 | 5 | 2 | t2396 | R | S | - |  |
| PC0018d-S0298 | 5 | 5 | 2 | t2396 | R | S | - |  |
| PC0018e-S0338 | 101 | 101 | 1 | NT | R | S | - |  |
| PC0019a-S0019 | 25 | 25 | 1 | NT | R | S | - |  |
| PC0019b-S0081 | 25 | 25 | 1 | new | R | S | - |  |
| PC0019c-S0130 | 25 | 25 | 1 | new | R | S | - |  |
| PC0019d-S0169 | 25 | 25 | 1 | new | R | S | - |  |
| PC0019e-S0272 | 25 | 25 | 1 | new | R | S | - |  |
| PC0019f-S0307 | 25 | 25 | 1 | new | R | S | - |  |
| PC0020-S0020 | 30 | 39 | 3 | t342 | R | S | - |  |
| PC0021a-S0021 | 5 | 5 | 2 | NT | R | S | - |  |
| PC0021b-S0208 | 5 | 5 | 2 | t002 | R | S | - |  |
| PC0022a-S0022 | 1 | 188 | 1 | t189 | R | S | - |  |
| PC0022b-S0247 | 1 | 188 | 1 | t189 | R | S | - |  |
| PC0023-S0023 | 97 | 97 | 1 | t267 | R | S | - |  |
| PC0024-S0024 | 30 | 30 | 3 | t021 | S | S | - |  |
| PC0025a-S0025 | 5 | 6 | 1 | t701 | R | S | - |  |
| PC0025b-S0281 | 5 | 6 | 1 | new | R | S | - |  |
| PC0026-S0026 | 8 | 8 | 1 | NT | R | S | - |  |
| PC0027a-S0027 | 45 | 508 | 1 | t026 | R | S | - |  |
| PC0027b-S0100 | 45 | 508 | 1 | t026 | R | S | - |  |
| PC0027c-S0206 | 45 | 508 | 1 | t026 | R | S | - |  |
| PC0028a-S0028 | 8 | 8 | 1 | t1476 | R | S | - |  |
| PC0028b-S0096 | 8 | 8 | 1 | t1476 | R | S | - |  |
| PC0028c-S0213 | 8 | 8 | 1 | t1476 | R | S | - |  |
| PC0028d-S0230 | 8 | 8 | 1 | t104 | R | S | - |  |
| PC0028e-S0238 | 8 | 8 | 1 | t104 | R | S | - |  |
| PC0028f-S0303 | 8 | 8 | 1 | t1476 | R | S | - |  |
| PC0029-S0029 | 1 | 188 | 1 | t189 | R | S | - |  |
| PC0030a-S0030 | 1 | 109 | 2 | t209 | R | S | - |  |
| PC0030b-S0149 | 1 | 109 | 2 | t209 | R | S | - |  |
| PC0030c-S0249 | 1 | 109 | 2 | t209 | R | S | - |  |
| PC0031a-S0031 | 5 | 5 | 2 | t4382 | R | S | - |  |
| PC0031b-S0152 | 5 | 5 | 2 | t2249 | R | S | - |  |
| PC0032a-S0032 | 5 | 5 | 2 | t002 | R | S | - |  |
| PC0032b-S0297 | 97 | 1179 | 1 | t267 | R | S | - |  |
| PC0032c-S0305 | 5 | 5 | 2 | t002 | R | S | - |  |
| PC0033a-S0033 | 5 | 6 | 1 | t2467 | R | S | - |  |
| PC0033b-S0061 | 5 | 6 | 1 | t2467 | R | S | - |  |
| PC0033c-S0265 | 5 | 6 | 1 | t2467 | S | S | - |  |
| PC0033d-S0266 | 5 | 6 | 1 | t2467 | R | S | - |  |
| PC0034a-S0034 | 5 | 6 | 1 | t10774 | R | S | - |  |
| PC0034b-S0142 | 5 | 6 | 1 | t10774 | R | S | - |  |
| PC0034c-S0204 | 5 | 6 | 1 | t10774 | R | S | - |  |
| PC0034d-S0283 | 5 | 6 | 1 | t10774 | R | S | - |  |
| PC0034e-S0284 | 5 | 6 | 1 | t10774 | R | S | - |  |
| PC0035-S0035 | 88 | 88 | 3 | t13712 | R | S | - |  |
| PC0036-S0036 | 1 | 188 | 1 | t189 | R | S | - |  |
| PC0037a-S0037 | 5 | 5 | 2 | t1215 | R | S | - |  |
| PC0037b-S0075 | 5 | 5 | 2 | t1215 | R | S | - |  |
| PC0037c-S0191 | 5 | 5 | 2 | t1215 | R | S | - |  |
| PC0037d-S0254 | 45 | 1165 | 1 | NT | R | S | - |  |
| PC0038a-S0038 | 30 | 3383 | 3 | t459 | R | S | - |  |
| PC0038b-S0312 | 45 | 45 | 1 | t065 | R | S | - |  |
| PC0039a-S0039 | 45 | 45 | 1 | t015 | R | S | - |  |
| PC0039b-S0099 | 45 | 45 | 1 | t015 | R | S | - |  |
| PC0039c-S0123 | 45 | 45 | 1 | NT | R | S | - |  |
| PC0039d-S0207 | 45 | 45 | 1 | t230 | R | S | - |  |
| PC0039e-S0294 | 45 | 45 | 1 | t015 | R | S | - |  |
| PC0040-S0040 | 5 | 5 | 2 | t2666 | R | S | - |  |
| PC0041-S0041 | 5 | 6 | 1 | t701 | R | S | - |  |
| PC0042-S0042 | 45 | 45 | 1 | NT | R | S | - |  |
| PC0043a-S0043 | 121 | 2276 | 4 | NT | R | S | - |  |
| PC0043b-S0168 | 121 | 2276 | 4 | t876 | R | S | - |  |
| PC0044-S0044 | 59 | 5228 | 1 | t216 | R | S | - |  |
| PC0045-S0045 | 8 | 72 | 1 | t6509 | R | S | - |  |
| PC0046-S0046 | 5 | 5 | 2 | t067 | S | S | - |  |
| PC0047-S0047 | 8 | 8 | 1 | t3912 | R | S | - |  |
| PC0048a-S0048 | 22 | 22 | 1 | t3287 | R | R;[IVh(2B)] | - |  |
| PC0048b-S0280 | 5 | 5189 | 2 | new | R | S | - |  |
| PC0049a-S0049 | 15 | 15 | 2 | t605 | R | S | - |  |
| PC0049b-S0131 | 15 | 15 | 2 | t774 | R | S | - |  |
| PC0049c-S0285 | 15 | 15 | 2 | t774 | R | S | - |  |
| PC0050a-S0050 | 1 | 188 | 1 | t189 | R | S | - |  |
| PC0050b-S0301 | 1 | 188 | 1 | t189 | R | S | - |  |
| PC0051a-S0051 | 22 | 22 | 1 | t6669 | R | S | - |  |
| PC0051b-S0069 | 22 | 22 | 1 | t6669 | R | S | - |  |
| PC0052-S0052 | 5 | 5 | 2 | t1265 | R | S | - |  |
| PC0053-S0053 | 45 | 45 | 1 | t065 | R | S | - |  |
| PC0054-S0054 | 1 | 109 | 2 | t209 | R | S | - |  |
| PC0055-S0055 | 97 | 97 | 1 | t4206 | S | S | - |  |
| PC0056a-S0056 | 45 | 5246 | 4 | t1081 | R | S | - |  |
| PC0056b-S0157 | 45 | 5246 | 4 | t1081 | R | S | - |  |
| PC0057a-S0057 | 5 | 6 | 1 | NT | R | S | - |  |
| PC0057b-S0121 | 5 | 6 | 1 | t5413 | R | S | - |  |
| PC0057c-S0299 | 5 | 6 | 1 | t5413 | R | S | - |  |
| PC0058ai-S0058 | 7 | 7 | 1 | NT | R | S | - |  |
| PC0058aii-S0225 | 8 | 72 | 1 | t2473 | R | S | - |  |
| PC0058aiii-S0224 | 7 | 7 | 1 | NT | R | S | - |  |
| PC0058b-S0083 | 7 | 7 | 1 | t091 | R | S | - |  |
| PC0058c-S0219 | 7 | 5247 | 1 | t1943 | R | S | - |  |
| PC0058d-S0400 | 7 | 7 | 1 | t1943 | R | S | - |  |
| PC0059a-S0059 | 8 | 8 | 1 | t2104 | R | S | - |  |
| PC0059b-S0078 | 8 | 8 | 1 | t2104 | R | S | - |  |
| PC0059c-S0089 | 30 | 30 | 3 | t338 | R | S | - |  |
| PC0059d-S0261 | 30 | 30 | 3 | t338 | R | S | - |  |
| PC0060a-S0060 | 30 | 2897 | 3 | t10176 | R | S | - |  |
| PC0060b-S0276 | 30 | 30 | 3 | t10176 | R | S | - |  |
| PC0060c-S0172 | 45 | 508 | 1 | t116 | R | S | - |  |
| PC0062-S0062 | 8 | 8 | 1 | t008 | S | S | - |  |
| PC0063-S0063 | 5 | 5 | 2 | t179 | R | S | - |  |
| PC0064-S0064 | 15 | 15 | 2 | t084 | R | S | - |  |
| PC0065-S0065 | 1 | 1 | 3 | t127 | R | S | + |  |
| PC0066-S0066 | 7 | 7 | 1 | t091 | R | S | - |  |
| PC0067-S0287 | 30 | 30 | 3 | t012 | R | S | - |  |
| PC0068a-S0068 | 30 | 30 | 3 | t363 | R | S | - |  |
| PC0068b-S0085 | 30 | 30 | 3 | t363 | R | S | - |  |
| PC0068c-S0125 | 30 | 30 | 3 | t363 | R | S | - |  |
| PC0068d-S0141 | 30 | 30 | 3 | t363 | R | S | - |  |
| PC0068e-S0201 | 5 | 5 | 2 | t179 | R | S | - |  |
| PC0068f-S0242 | 30 | 30 | 3 | t363 | R | S | - |  |
| PC0068g-S0391 | 30 | 30 | 3 | t363 | R | S | - |  |
| PC0070a-S0070 | 15 | 15 | 2 | t346 | R | S | - |  |
| PC0070b-S0112 | 15 | 15 | 2 | t346 | R | S | - |  |
| PC0071a-S0071 | 8 | 8 | 1 | t3912 | R | S | - |  |
| PC0071b-S0259 | 8 | 8 | 1 | t14519 | R | S | - |  |
| PC0072-S0072 | 5 | 5 | 2 | t002 | R | S | - |  |
| PC0073a-S0073 | 8 | 72 | 1 | t148 | R | S | - |  |
| PC0073b-S0167 | 8 | 72 | 1 | t148 | R | S | - |  |
| PC0073c-S0210 | 8 | 72 | 1 | t2473 | R | S | - |  |
| PC0074a-S0074 | 30 | 39 | 3 | t2479 | R | S | - |  |
| PC0074b-S0117 | 30 | 39 | 3 | t2479 | R | S | - |  |
| PC0074c-S0186 | 30 | 39 | 3 | t2479 | R | S | - |  |
| PC0075-S0288 | 5 | 149 | 2 | t002 | R | R;[IVb(2B)] | - |  |
| PC0076a-S0076 | 1 | 1 | 3 | t922 | R | S | - |  |
| PC0076b-S0140 | 1 | 1 | 3 | t922 | R | S | - |  |
| PC0077-S0077 | 398 | 291 | 1 | t1991 | S | S | - |  |
| PC0079-S0079 | 8 | 8 | 1 | t008 | R | S | - |  |
| PC0080-S0190 | 1 | 109 | 2 | t209 | R | S | - |  |
| PC0081-S0291 | 15 | 15 | 2 | t084 | R | S | - |  |
| PC0082a-S0082 | 88 | 88 | 3 | t186 | R | S | - |  |
| PC0082b-S0234 | 88 | 88 | 3 | t237 | R | S | - |  |
| PC0083-S0292 | 1 | 109 | 2 | t209 | R | S | - |  |
| PC0084-S0084 | 5 | 5 | 2 | NT | R | S | - |  |
| PC0085-S0293 | 30 | 5482 | 3 | t382 | R | S | - |  |
| PC0086a-S0086 | 5 | 5 | 2 | NT | R | S | - |  |
| PC0086b-S0390 | 5 | 5 | 2 | t450 | S | S | - |  |
| PC0087-S0295 | 1 | 188 | 1 | t189 | R | S | - |  |
| PC0088a-S0088 | 30 | 5227 | 3 | t3018 | R | S | - |  |
| PC0088b-S0264 | 30 | 5227 | 3 | t3018 | R | S | - |  |
| PC0089-S0296 | 30 | 30 | 3 | t012 | R | S | - |  |
| PC0090a-S0090 | 8 | 8 | 1 | t622 | R | S | - |  |
| PC0090b-S0233 | 5 | 5 | 2 | t2666 | R | S | - |  |
| PC0090c-S0289 | 5 | 5 | 2 | t2666 | R | S | - |  |
| PC0091-S0091 | 97 | 5244 | 1 | t267 | S | S | - |  |
| PC0092a-S0092 | 5 | 5 | 2 | NT | R | S | - |  |
| PC0092b-S0211 | 5 | 5 | 2 | t539 | R | S | - |  |
| PC0092c-S0252 | 5 | 5 | 2 | t539 | R | S | - |  |
| PC0093-S0093 | 30 | 30 | 3 | t338 | R | S | - |  |
| PC0094-S0094 | 5 | 5 | 2 | NT | R | S | - |  |
| PC0095a-S0095 | 45 | 508 | 1 | t583 | S | S | - |  |
| PC0095b-S0109 | 45 | 508 | 1 | t728 | S | S | - |  |
| PC0095c-S0135 | 45 | 508 | 1 | t302 | S | S | - |  |
| PC0095d-S0246 | 45 | 508 | 1 | t302 | S | S | - |  |
| PC0095e-S0235 | 45 | 508 | 1 | t302 | S | S | - |  |
| PC0097a-S0097 | 15 | 15 | 2 | t673 | R | S | - |  |
| PC0097b-S0180 | 15 | 15 | 2 | t10602 | R | S | - |  |
| PC0098-S0098 | 121 | 2276 | 4 | t876 | R | S | - |  |
| PC0099-S0306 | 45 | 5484 | - | t095 | R | S | - |  |
| PC0100-S0308 | 5 | 5 | 2 | t1265 | S | S | - |  |
| PC0101-S0309 | 15 | 15 | 2 | t803 | R | S | - |  |
| PC0102-S0102 | 5 | 5 | 2 | t19770 | S | S | - |  |
| PC0103a-S0103 | 1 | 188 | 1 | t189 | R | S | - |  |
| PC0103b-S0194 | 1 | 188 | 1 | t189 | R | S | - |  |
| PC0104-S0310 | 88 | 88 | 3 | t11285 | R | S | - |  |
| PC0105a-S0105 | 45 | 45 | 1 | t728 | S | S | - |  |
| PC0105b-S0217 | 1 | 109 | 2 | t209 | S | S | - |  |
| PC0105c-S0199 | 45 | 508 | 1 | t230 | R | S | - |  |
| PC0105d-S0273 | 45 | 508 | 1 | NT | R | S | - |  |
| PC0106-S0106 | 5 | 5189 | 2 | t1265 | R | S | - |  |
| PC0107-S0107 | 5 | 5 | 2 | t1265 | R | S | - |  |
| PC0108-S0108 | 1 | 1 | 3 | t114 | R | S | - |  |
| PC0109a-S0311 | 1 | 1 | 3 | t559 | R | S | - |  |
| PC0109b-S0392 | 1 | 1 | 3 | t559 | R | S | - |  |
| PC0110a-S0110 | 45 | 45 | 1 | t3000 | R | S | - |  |
| PC0110b-S0119 | 45 | 45 | 1 | t3000 | R | S | - |  |
| PC0110c-S0182 | 45 | 45 | 1 | t3000 | R | S | - |  |
| PC0110d-S0282 | 45 | 45 | 1 | t3000 | R | S | - |  |
| PC0111-S0111 | 45 | 5231 | 1 | t230 | R | S | - |  |
| PC0113a-S0113 | 5 | 5245 | 2 | NT | R | S | - |  |
| PC0113b-S0237 | 5 | 5245 | 2 | NT | R | S | - |  |
| PC0114a-S0114 | 30 | 5230 | 3 | t018 | R | S | - |  |
| PC0114b-S0236 | 30 | 5248 | 3 | NT | R | S | - |  |
| PC0115-S0115 | 88 | 78 | 3 | t4385 | S | S | - |  |
| PC0116-S0116 | 25 | 25 | 1 | t436 | R | S | - |  |
| PC0118-S0118 | 30 | 30 | 3 | NT | R | S | - |  |
| PC0120-S0120 | 15 | 15 | 2 | t1361 | R | S | - |  |
| PC0122-S0122 | 5 | 5 | 2 | t1265 | R | S | - |  |
| PC0124a-S0124 | 97 | 5232 | 1 | t359 | S | S | - |  |
| PC0124b-S0248 | 97 | 97 | 1 | t359 | S | S | - |  |
| PC0126-S0126 | 121 | 2276 | 4 | t876 | R | S | - |  |
| PC0127-S0127 | 5 | 5 | 2 | t002 | R | S | - |  |
| PC0128a-S0128 | 5 | 5 | 2 | t002 | S | S | - |  |
| PC0128b-S0164 | 5 | 5 | 2 | new | S | S | - |  |
| PC0129-S0129 | 45 | 45 | 1 | t040 | R | S | - |  |
| PC0132a-S0132 | 5 | 5 | 2 | t242 | R | S | - |  |
| PC0132b-S0304 | 5 | 5 | 2 | t002 | R | S | - |  |
| PC0133a-S0133 | 7 | 7 | 1 | t1943 | R | S | - |  |
| PC0133b-S0250 | 7 | 7 | 1 | t091 | R | S | - |  |
| PC0134-S0134 | 5 | 5 | 2 | t002 | S | S | - |  |
| PC0136-S0136 | 5 | 5 | 2 | t1265 | R | S | - |  |
| PC0137-S0137 | 88 | 88 | 3 | new | R | S | - |  |
| PC0138a-S0138 | 5 | 5 | 2 | t548 | R | S | - |  |
| PC0138b-S0244 | 5 | 5 | 2 | NT | R | S | - |  |
| PC0139-S0139 | 5 | 5233 | 2 | t071 | R | S | - |  |
| PC0143-S0143 | 1 | 188 | 1 | t189 | S | S | - |  |
| PC0144-S0144 | 8 | 5234 | 1 | t008 | R | S | - |  |
| PC0145-S0145 | 88 | 88 | 3 | t4701 | R | S | - |  |
| PC0146-S0146 | 45 | 5235 | 1 | t026 | R | S | - |  |
| PC0147-S0147 | 8 | 5236 | 1 | NT | R | S | - |  |
| PC0148-S0148 | 30 | 34 | 3 | t4442 | R | S | - |  |
| PC0150-S0150 | 15 | 15 | 2 | NT | R | S | - |  |
| PC0151a-S0151 | 121 | 121 | 4 | t5072 | R | S | - |  |
| PC0151b-S0239 | 121 | 121 | 4 | t5072 | R | S | - |  |
| PC0153-S0153 | 8 | 8 | 1 | t648 | R | S | - |  |
| PC0154a-S0154 | 5 | 5 | 2 | t1265 | R | S | - |  |
| PC0154b-S0263 | 121 | 2276 | 4 | t876 | R | S | - |  |
| PC0155a-S0155 | 1 | 1 | 3 | t127 | R | S | - |  |
| PC0155b-S0277 | 1 | 1 | 3 | t127 | R | S | - |  |
| PC0155c-S0300 | 5 | 6 | 1 | t2467 | R | S | - |  |
| PC0156-S0156 | 1 | 109 | 2 | t209 | R | S | - |  |
| PC0159-S0159 | 5 | 5 | 2 | t002 | R | S | - |  |
| PC0160-S0160 | 97 | 97 | 1 | t267 | R | S | - |  |
| PC0161-S0161 | 30 | 30 | 3 | t122 | R | S | - |  |
| PC0162-S0162 | 398 | 398 | 1 | t3625 | R | S | - |  |
| PC0170-S0170 | 30 | 30 | 3 | t012 | R | S | - |  |
| PC0174-S0174 | 5 | 5 | 2 | t002 | R | S | - |  |
| PC0175a-S0175 | 30 | 30 | 3 | t4242 | R | S | - |  |
| PC0175b-S0255 | 30 | 30 | 3 | t4242 | R | S | - |  |
| PC0175c-S0337 | 30 | 30 | 3 | t4242 | R | S | - |  |
| PC0176a-S0176 | 30 | 30 | 3 | t012 | R | S | - |  |
| PC0176b-S0253 | 30 | 30 | 3 | t012 | R | S | - |  |
| PC0177ai-S0177 | 1 | 188 | 1 | t189 | R | S | - |  |
| PC0177aii-S0226 | 5 | 6 | 1 | NT | R | S | - |  |
| PC0177b-S0274 | 5 | 6 | 1 | t9476 | R | S | - |  |
| PC0177c-S0165 | 5 | 6 | 1 | t9476 | R | S | - |  |
| PC0178a-S0178 | 88 | 5249 | 3 | t186 | R | S | - |  |
| PC0178b-S0240 | 88 | 5249 | 3 | t186 | R | S | - |  |
| PC0179i-S0179 | 30 | 30 | 3 | t1504 | R | S | - |  |
| PC0179ii-S0227 | 30 | 30 | 3 | t1504 | R | S | - |  |
| PC0179iii-S0228 | 30 | 30 | 3 | t1504 | R | S | - |  |
| PC0181-S0181 | 5 | 5 | 2 | t002 | R | S | - |  |
| PC0183-S0183 | 30 | 5237 | 3 | t2018 | R | S | - |  |
| PC0184-S0184 | 5 | 5 | 2 | new | R | S | - |  |
| PC0185-S0185 | 5 | 5 | 2 | NT | R | S | - |  |
| PC0187a-S0187 | 30 | 3383 | 3 | t459 | R | S | - |  |
| PC0187b-S0290 | 30 | 3383 | 3 | t459 | R | S | - |  |
| PC0189a-S0189 | 30 | 39 | 3 | t638 | R | S | - |  |
| PC0189b-S0218 | 30 | 39 | 3 | t638 | R | S | - |  |
| PC0189c-S0231 | 30 | 39 | 3 | t638 | R | S | - |  |
| PC0192-S0192 | 45 | 45 | 1 | t371 | R | S | - |  |
| PC0193a-S0193 | 15 | 15 | 2 | t1877 | R | S | - |  |
| PC0193b-S0251 | 15 | 15 | 2 | t346 | R | S | - |  |
| PC0196-S0196 | 30 | 34 | 3 | t089 | R | S | - |  |
| PC0197-S0197 | 30 | 30 | 3 | t338 | R | S | - | |
| PC0198a-S0198 | 5 | 5223 | 2 | t002 | S | S | - |  |
| PC0198b-S0220 | 5 | 5223 | 2 | t062 | S | S | - |  |
| PC0202-S0202 | 5 | 5 | 2 | NT | R | R;[V(5C2&5)] | - |  |
| PC0203-S0203 | 121 | 2276 | 4 | t876 | R | S | - |  |
| PC0205-S0205 | 45 | 45 | 1 | t371 | R | S | - |  |
| PC0209-S0209 | 5 | 5 | 2 | t045 | R | S | - |  |
| PC0212-S0212 | 5 | 5 | 2 | t002 | R | R;[IVb(2B)] | - |  |
| PC0214-S0214 | 30 | 39 | 3 | t11025 | R | S | - |  |
| PC0215a-S0215 | 1 | 5238 | 1 | t189 | R | S | - |  |
| PC0215b-S0229 | 1 | 188 | 1 | t189 | R | S | - |  |
| PC0221-S0221 | 8 | 72 | 1 | t12138 | R | S | - |  |
| PC0222a-S0222 | 88 | 88 | 3 | t448 | R | S | - |  |
| PC0222b-S0262 | 45 | 5250 | 1 | t132 | R | S | - |  |
| PC0223-S0223 | 1 | 188 | 1 | t189 | R | S | - |  |
| PC0232-S0232 | 97 | 97 | 1 | t267 | R | S | - |  |
| PC0241-S0241 | 5 | 5 | 2 | NT | R | S | - |  |
| PC0243-S0243 | 8 | 72 | 1 | t3169 | R | S | - |  |
| PC0245-S0245 | 15 | 15 | 2 | t085 | R | S | - |  |
| PC0249-S0388 | 8 | 8 | 1 | t121 | R | S | - |  |
| PC0250-S0389 | 5 | 5 | 2 | t1265 | R | S | - |  |
| PC0256-S0256 | 121 | 2276 | 4 | t876 | R | S | - |  |
| PC0257-S0257 | 20 | 20 | 1 | t2451 | R | S | - |  |
| PC0258-S0258 | 20 | 20 | 1 | t164 | S | S | - |  |
| PC0260-S0260 | 5 | 5 | 2 | t002 | S | S | - |  |
| PC0267ai-S0267 | 1 | 1 | 3 | t127 | R | S | - |  |
| PC0267aii-S0268 | 7 | 7 | 1 | t091 | R | S | - |  |
| PC0267b-S0275 | 1 | 1 | 3 | t127 | R | S | - |  |
| PC0267c-S0286 | 45 | 45 | 1 | NT | R | S | - |  |
| PC0269-S0269 | 5 | 5 | 2 | new | R | S | - |  |
| PC0270-S0270 | 20 | 20 | 1 | t693 | S | S | - |  |
| PC0271-S0271 | 5 | 5251 | 1 | t701 | S | S | - |  |
| PN0096-S0302 | 5 | 5483 | 2 | t002 | R | R;[IV(2B)] | - |  |
| PN0112-S0313 | 121 | 121 | 4 | t645 | R | S | + |  |
| PN0117-S0314 | 15 | 15 | 2 | t085 | R | S | - |  |
| PN0119a-S0315 | 30 | 30 | 3 | t4557 | R | R;[IVc(2B)] | + |  |
| PN0119b-S0325 | 30 | 30 | 3 | t4557 | R | R;[IVc(2B)] | + |  |
| PN0121-S0316 | 5 | 5485 | 2 | t179 | R | S | - |  |
| PN0123-S0317 | 25 | 25 | 1 | t287 | R | S | + |  |
| PN0125-S0318 | 5 | 5 | 2 | t088 | S | S | - |  |
| PN0130-S0319 | 1 | 1 | 3 | t127 | R | S | + |  |
| PN0131-S0320 | 121 | 2276 | 4 | t876 | R | S | - |  |
| PN0135-S0321 | 30 | 39 | 3 | t2271 | S | S | - |  |
| PN0140-S0322 | 5 | 6 | 1 | t304 | R | S | - |  |
| PN0141-S0323 | 5 | 5 | 2 | t1265 | R | S | - |  |
| PN0142-S0324 | 8 | 72 | 1 | t3169 | R | S | - |  |
| PN0149-S0326 | 1 | 1 | 3 | t127 | S | S | + |  |
| PN0152-S0327 | 121 | 5486 | 4 | t159 | R | S | + |  |
| PN0157-S0328 | 1 | 1 | 3 | t127 | S | S | + |  |
| PN0158-S0329 | 5 | 5 | 2 | NT | R | S | - |  |
| PN0163-S0330 | 121 | 2276 | 4 | t876 | R | S | - |  |
| PN0164-S0331 | 15 | 15 | 2 | t335 | R | S | - |  |
| PN0165a-S0332 | 22 | 22 | 1 | t005 | R | R;[IVa(2B)] | + |  |
| PN0165b-S0363 | ST5491 | 5491 | 3 | t5925 | R | S | - |  |
| PN0166-S0333 | 88 | 88 | 3 | t786 | R | S | - |  |
| PN0167-S0334 | 96 | 1930 | 3 | NT | R | R;[IVa(2B)] | + |  |
| PN0168-S0335 | 30 | 30 | 3 | new | R | S | - |  |
| PN0169-S0336 | 5 | 5 | 2 | t548 | R | S | - |  |
| PN0171a-S0339 | 121 | 121 | 4 | t159 | R | S | + |  |
| PN0171b-S0341 | 121 | 121 | 4 | t159 | R | S | + |  |
| PN0173-S0340 | 88 | 78 | 3 | t7558 | R | R[IVa(2B)] | - |  |
| PN0180-S0342 | 5 | 5 | 2 | t548 | R | S | - |  |
| PN0182-S0343 | 5 | 2304 | 2 | NT | R | S | - |  |
| PN0186-S0344 | 97 | 97 | 1 | t1236 | R | S | - |  |
| PN0188a-S0345 | 8 | 72 | 1 | t148 | R | S | - |  |
| PN0188b-S0378 | 1 | 1 | 3 | t127 | R | S | - |  |
| PN0190-S0346 | 15 | 582 | 2 | t084 | R | S | - |  |
| PN0191-S0347 | 8 | 8 | 1 | t008 | R | S | - |  |
| PN0194-S0348 | 5 | 5 | 2 | t105 | R | S;[IVc(2B)] | + |  |
| PN0199-S0349 | 93 | 93 | 3 | NT | S | S;[IVa(2B)] | + |  |
| PN0200ai-S0350 | 93 | 93 | 3 | t267 | R | S | + |  |
| PN0200aii-S0402 | 88 | 78 | 3 | t2393 | R | S;[IVa(2B)] | - |  |
| PN0201-S0351 | 93 | 93 | 3 | NT | R | S;[IVa(2B)] | + |  |
| PN0204-S0352 | 93 | 93 | 3 | t202 | R | S;[IVa(2B)] | + |  |
| PN0206-S0353 | 30 | 30 | 3 | t019 | R | R;[IVc(2B)] | + |  |
| PN0207a-S0354 | 20 | 1281 | 1 | t5587 | R | S | - |  |
| PN0207b-S0393 | 88 | 88 | 3 | t786 | R | S | - |  |
| PN0208-S0355 | 121 | 121 | 4 | t159 | R | S | - |  |
| PN0210-S0356 | 93 | 93 | 3 | t3949 | R | R;[IV(2B)] | + |  |
| PN0211-S0357 | 30 | 30 | 3 | t019 | R | R;[IVc(2B)] | + |  |
| PN0213a-S0358 | 121 | 2276 | 4 | t876 | R | S | - |  |
| PN0213b-S0359 | 121 | 2276 | 4 | t876 | R | S | - |  |
| PN0216-S0360 | 5 | 6 | 1 | t701 | S | S | - |  |
| PN0217-S0361 | 30 | 5487 | 3 | t019 | S | S | + |  |
| PN0218-S0362 | 93 | 93 | 3 | NT | R | R;[IVa(2B)] | + |  |
| PN0219-S0364 | 15 | 15 | 2 | NT | R | S | - |  |
| PN0220-S0365 | 15 | 15 | 2 | NT | R | S | - |  |
| PN0224-S0366 | 30 | 34 | 3 | t365 | R | S | - |  |
| PN0225-S0367 | 1 | 188 | 1 | t2883 | R | S | - |  |
| PN0226-S0368 | 1 | 1 | 3 | t127 | R | S | + |  |
| PN0227-S0369 | 30 | 30 | 3 | t021 | R | S | - |  |
| PN0228-S0370 | 1 | 1 | 3 | t127 | R | S | + |  |
| PN0229-S0371 | 30 | 30 | 3 | NT | R | R;[IVc(2B)] | + |  |
| PN0230-S0372 | 30 | 30 | 3 | t012 | R | S | - |  |
| PN0231-S0373 | 121 | 121 | 4 | t159 | R | S | + |  |
| PN0233-S0374 | 93 | 93 | 3 | NT | R | R;[IVa(2B)] | + |  |
| PN0234-S0375 | 15 | 15 | 2 | t346 | R | S | - |  |
| PN0235-S0376 | 121 | 121 | 4 | t162 | R | S | + |  |
| PN0236-S0377 | 398 | 398 | 1 | t1451 | S | S | - |  |
| PN0237-S0379 | 121 | 121 | 4 | t645 | R | S | + |  |
| PN0238-S0380 | 1 | 1 | 3 | t127 | R | S | + |  |
| PN0239-S0381 | 121 | 121 | 4 | t269 | R | S | + |  |
| PN0240-S0382 | 1 | 2990 | 2 | NT | R | S | + |  |
| PN0242-S0383 | 45 | 508 | 1 | t487 | R | S | - |  |
| PN0244-S0384 | 121 | 2276 | 4 | t876 | R | S | - |  |
| PN0246-S0385 | 15 | 15 | 2 | t085 | R | S | - |  |
| PN0247-S0386 | 121 | 121 | 4 | t1077 | R | S | + |  |
| PN0248-S0387 | 45 | 508 | 1 | t015 | R | S | - |  |
| PN0251-S0394 | 5 | 5 | 2 | t1265 | R | S | - |  |
| PN0252-S0395 | 5 | 5 | 2 | NT | S | S | - |  |
| PN0253-S0396 | 97 | 97 | 1 | t267 | R | R;[IVa(2B)] | - |  |
| PN0254-S0397 | 1 | 109 | 2 | t3745 | R | S | - |  |
| PN0255-S0398 | 1 | 1 | 3 | t127 | R | S | + |  |
| PN0259-S0399 | 121 | 121 | 4 | t162 | R | S | + |  |
| PN0261-S0401 | 30 | 5488 | 3 | t710 | R | S | - |  |

MLST – multilocus sequence type, CC – clonal complex, NT – not typeable, R – resistant, S – susceptible, + present, - absent.
