# Supplementary material for: Limited evidence of patient-to-patient transmission of Staphylococcus aureus strains between children with cystic fibrosis, Queensland, Australia
Source: PLoS One. 2022 Oct 7;17(10):e0275256. doi: 10.1371/journal.pone.0275256 (PMC9543978; doi:10.1371/journal.pone.0275256)
Supplement: S2 Table — MLST, agr and spa types and number of cases for the major clonal complexes. (DOCX) [file pone.0275256.s002.docx]

**Title:** Limited evidence of patient-to-patient transmission of *Staphylococcus aureus* strains between children with cystic fibrosis, Queensland, Australia.

**Journal:** PLOS ONE

**Authors**: Sharon L Biggs^1^, Amy V Jennison^2^, Haakon Bergh^3^, Rikki Graham^2^, Graeme Nimmo^4^, David Whiley^1,3*^

^1^School of Medicine, The University of Queensland, UQ Centre for Clinical Research (UQCCR), Herston, Queensland 4029, Australia; ^2^Public and Environmental Health, Forensic and Scientific Services, Coopers Plains, Queensland 4108, Australia; ^3^Pathology Queensland Central Laboratory, Herston, Queensland, 4029, Australia; ^4^School of Medicine, Griffith University, Gold Coast, Queensland 4215, Australia.

*Corresponding author: Assoc Professor David Whiley, The University of Queensland, UQ Centre for Clinical Research (UQCCR), Herston, Queensland 4029, Australia.

Email: [d.whiley@uq.edu.au](mailto:d.whiley@uq.edu.au)

Phone: +61 7 3346 5053

**Supplementary Information SI2**

**Table SI2** Summary of the major Clonal Complexes by ST, *agr* and *spa* types

| **CC** | **MLST** | ***agr*** | ***spa*** | **Cases (n=287)** |
| --- | --- | --- | --- | --- |
| 5 | 5 | 2 | t002, t010, t045, t062, t067, t088, t105, t179, t242, t450, t539, t548, t653, t1215, t1265, t2249, t2396, t2666, t4382, t19770, NT | 51 |
|  | 6 | 1 | t10774, t2467, t5413, t701, t9476, NT | 9 |
| 30 | 30 | 3 | t012, t019, t021, t10176, t122, t1504, t338, t363, t4242, t4557, t4733, NT | 22 |
|  | 34 | 3 | t089, t365, t4442 | 3 |
|  | 39 | 3 | t11025, t2479, t342, t638, t2271 | 5 |
| 1 | 1 | 3 | t114, t127, t14508, t559, t922 | 16 |
|  | 109 | 2 | t209, t3745 | 7 |
|  | 188 | 1 | t189, t2883 | 11 |
| 45 | 45 | 1 | t015, t040, t065, t230, t3000, t371, t728 | 10 |
|  | 508 | 1 | t015, t487, t026, t116, t230, t302, t583, t728 | 6 |
| 8 | 8 | 1 | t008, t104, t121, t14519, t1476, t2104, t3912, t622, t648, NT | 12 |
|  | 72 | 1 | t12138, t148, t2473, t3169, t6509 | 7 |
| 15 | 15 | 2 | t084, t085, t335, t346, t10602, t12818, t1361, t1877, t605, t673, t774, t803, NT | 19 |
| 88 | 78 | 3 | t2393, t4385, t7558, NT | 4 |
|  | 88 | 3 | t186, t237, t448, t786, t11285, t13712, t4701, NT | 8 |
| 97 | 97 | 1 | t267, t359, t1236, t4206 | 7 |
| 121 | 121 | 4 | t1077, t159, t162, t269, t645, t5072 | 10 |
| 7 | 7 | 1 | t091, t1943, NT | 4 |
| 20 | 20 | 1 | t164, t2451, t693 | 4 |
| 398 | 398 | 1 | t1184, t1451, t3625 | 3 |
| 101 | 101 | 1 | t2078, NT | 2 |
| 22 | 22 | 1 | t005, t3287, t6669 | 3 |
| 25 | 25 | 1 | t287, t436, NT | 3 |
| 93 | 93 | 3 | t202, t267, t3949,NT | 7 |

NT – non-typeable or new *spa* type. Patient cases: multiple isolates from the same patients with the same Clonal Complex and MLST - counted as one case; different Clonal Complexes and/or MLST - counted as separate cases.
